# Application of Novel Polymorphic Microsatellite Loci Identified in the Korean Pacific Abalone (*Haliotis diversicolor supertexta* (Haliotidae)) in the Genetic Characterization of Wild and Released Populations

**DOI:** 10.3390/ijms130910750

**Published:** 2012-08-28

**Authors:** Hye Suck An, Jang Wook Lee, Seong Wan Hong

**Affiliations:** 1 Biotechnology Research Division, National Fisheries Research and Development Institute, Busan 619-705, Korea; E-Mail: lee9952@nfrdi.go.kr; 2 Jeju Province Fisheries Research Institute, Jeju 699-814, Korea; E-Mail: hsw4905@korea.kr

**Keywords:** Pacific abalone, *Haliotis diversicolor supertexta*, *Haliotis diversicolor diversicolor*, microsatellite loci, next-generation sequencing, genetic diversity

## Abstract

The small abalone, *Haliotis diversicolor supertexta*, of the family Haliotidae, is one of the most important species of marine shellfish in eastern Asia. Over the past few decades, this species has drastically declined in Korea. Thus, hatchery-bred seeds have been released into natural coastal areas to compensate for the reduced fishery resources. However, information on the genetic background of the small abalone is scarce. In this study, 20 polymorphic microsatellite DNA markers were identified using next-generation sequencing techniques and used to compare allelic variation between wild and released abalone populations in Korea. Using high-throughput genomic sequencing, a total of 1516 (2.26%; average length of 385 bp) reads containing simple sequence repeats were obtained from 86,011 raw reads. Among the 99 loci screened, 28 amplified successfully, and 20 were polymorphic. When comparing allelic variation between wild and released abalone populations, a total of 243 different alleles were observed, with 18.7 alleles per locus. High genetic diversity (mean heterozygosity = 0.81; mean allelic number = 15.5) was observed in both populations. A statistical analysis of the fixation index (*F*_ST_) and analysis of molecular variance (AMOVA) indicated limited genetic differences between the two populations (*F*_ST_ = 0.002, *p* > 0.05). Although no significant reductions in the genetic diversity were found in the released population compared with the wild population (*p* > 0.05), the genetic diversity parameters revealed that the seeds released for stock abundance had a different genetic composition. These differences are likely a result of hatchery selection and inbreeding. Additionally, all the primer pair sets were effectively amplified in another congeneric species, *H. diversicolor diversicolor*, indicating that these primers are useful for both abalone species. These microsatellite loci may be valuable for future aquaculture and population genetic studies aimed at developing conservation and management plans for these two abalone species.

## 1. Introduction

Pacific abalones are gastropod mollusks that are highly valued, especially in eastern Asia. *Haliotis diversicolor supertexta*, which belongs to the Haliotidae family, is a small (maximum shell length of 10–12 cm) abalone that varies in color and is a commercially important species distributed throughout the southern coasts of Korea, Japan, China and Taiwan [1,2]. In Korea, fisheries operate primarily throughout the coastal areas of Jeju Island. The annual catch of this abalone has decreased drastically since its peak in 1997, and the commercial catch has declined by 90% over a 10-year period, reaching a historic low of approximately 12 tons in 2006 [3]. To compensate for these reduced fishery resources, enhancement practices have been initiated by the Jeju Province Fisheries Institute (JPFI) that involve the release of hatchery stock into natural coastal areas and have been ongoing since 2005 [4]. However, these enhancement practices raise concerns regarding the genetic diversity of artificial abalones; indeed, the reduction in genetic diversity has been observed in most hatchery stocks can be explained by genetic drift phenomena or founding effects [5]. Thus, genetic monitoring of hatchery stocks and natural populations is recommended to help preserve the genetic variation in natural populations [6]. In recent years, increasing concerns regarding the status of this abalone stock due to overfishing and marine pollution have been raised [4]. Knowledge regarding the genetic variability and the patterns of the stock structure is a prerequisite for developing effective fishery conservation strategies as well as management and remediation efforts [7]. Thus, a better understanding of the genetic structure of *H. diversicolor supertexta* would aid in the development of a more effective fishery management strategy for this species. However, no information is available on the genetic diversity of wild and released populations of *H. diversicolor supertexta*.

Monitoring genetic variation among marine resources necessitates the development of genetic markers. Among the available genetic markers, simple sequence repeats (SSRs) or microsatellites (MS) are an extremely valuable tool for population genetic studies as well as conservation and management of genetic resources because of their useful properties, which include high levels of polymorphism, codominant inheritance and good reproducibility [8–10]. MS markers have been developed for *H. diversicolor* [11–13]; however, most of these markers have relatively few alleles and low heterozygosity, and there are no reports about its ability to evaluate the genetic structure of a population. Furthermore, using MS loci across closely related species is often complicated by the occurrence of null alleles, and many loci may prove to be of very limited use as polymorphic markers even when used in a sibling species [14,15]. Therefore, a large number of species-specific MS for *H. diversicolor supertexta* must be developed and screened to identify a suite of loci that are powerful and efficient for conducting further population genetic analyses, including assignment tests, pedigree analyses and mapping studies.

Historically, MS markers were developed by screening small-insert genomic DNA libraries or repeat-enriched libraries [16]. These time-consuming and expensive procedures have been limited by their dependence on the repeat motif of the probes used [17]. There are numerous reports that MS isolation has failed or resulted in a very low yield of polymorphic markers [18,19]. However, recent advances in the technology and accessibility of high-throughput genomic sequencing, next-generation sequencing platforms, such as the 454 GS-FLX platform (Roche Applied Science), are providing a much more efficient and cost-effective method for the acquisition of genetic markers (including MS) in organisms for which adequate databases are not currently available [20]. There are increasing reports employing this new technology in the effective development of MS markers in many taxa, including marine organisms [21–24].

In the present study, we developed 20 novel polymorphic MS primer sets for *H. diversicolor supertexta* using 454 GS-FLX pyrosequencing, and we examined the genetic variability at these loci in a wild and released populations of this species. Additionally, the applicability of these markers in another congener species, *Haliotis diversicolor diversicolor*, was evaluated via cross-species amplification experiments. The polymorphic MS markers described here will be useful in future genetic studies aimed at understanding the genetic status and facilitating the conservation of two Pacific abalones, *H. diversicolor supertexta* and *H. diversicolor diversicolor*, in Korea.

## 2. Results and Discussion

### 2.1. 454 Sequencing Results

The raw 454 sequence data from a 1/8 plate run included 26.9 Mbp containing 86,011 reads or sequences with an average length of 314.1 bp (maximum: 733 bp, minimum: 40 bp). Millions of base pairs of genomic sequence would be useful for both MS-related and MS-unrelated research [25], and the raw sequences could be assembled into contigs. A total of 5898 reads (approximately 6.9%) were assembled into 1278 contigs with an average length of 391 bp (maximum: 4835 bp, minimum: 100 bp), leaving 65,632 singletons. The mean length of these 66,910 sequences (1278 contigs plus 65,632 singletons) was 385 bp which was slightly longer than that of the raw sequences (385 bp *vs*. 314.1 bp). This process eliminates repetitive sequences and creates longer reads. We obtained a longer average read length compared with previous studies, with average read lengths of 369 bp in the water strider *Gerris incognitus* [21], 215 bp in the copperhead snake *Agkistrodon contortrix* [26] and 112 bp in the heavy-footed moa *Pachyornis elephantopus* [27]. Longer reads increase the likelihood of detecting loci with a greater number of repeats, which are expected to be more polymorphic, as well as the probability of detecting MS repeats and suitable primers within a single read [28]. The length of contigs generated based on short sequence reads depends on the depth of genome coverage [29]. Therefore, to develop a more comprehensive MS marker set via de novo sequencing, a sufficient depth of genome coverage is needed [22].

### 2.2. Isolation of Microsatellite Loci

Of the 66,910 unique sequences, 1516 (2.26%) contained simple sequence repeats, and 1143 (75.4%) contained a minimum of five di-, tri- or tetra-nucleotide repeat motifs, which were suitable for use as polymorphic MS markers. Motifs containing five to six repeats were the most abundant (78.6%), followed by seven to nine repeat motifs (17.7%) and motifs with more than ten repeats (3.7%). Among these, 244 sequences with a minimum of seven di-, tri- or tetra-nucleotide repeat motifs were used to develop MS primers. To design the primers, sequences that were of adequate length (more than 300 bp) and unique sequences flanking the MS array (minimum of 100 bases) were selected. Thus, 99 MS loci (32 di-, 22 tri- and 45 tetra- to hexa-nucleotides) were selected for subsequent polymorphism screening. Of these 99 MS loci, 28 (28.3%; seven di-, six tri- and 17 tetra- to hexa-nucleotides) were amplified successfully (as viewed on agarose gel) in the initial evaluation of the MS primers. The remaining 51 primers did not generate the desired amplification products in any of the eight samples despite retesting under modified PCR conditions. Additionally, amplifications of 20 loci produced faint or inconsistent bands, which may have been due to nonspecific PCR amplification. Further screening revealed that 20 (20.2%) loci were polymorphic in the eight *H. diversicolor supertexta* samples. The primer sequences, repeat motifs, annealing temperatures, fluorescent labels and GenBank accession numbers for the 20 new MS loci are summarized in Table 1.

The development of polymorphic MS markers using 454 sequencing technology is an outstanding new method. An increasing number of studies are using this technique to identify MS because it is less expensive and less time-consuming than traditional methods [25]. Using 454 pyrosequencing, we tested 99 primer pairs and characterized 20 polymorphic MS loci in *H. diversicolor supertexta*. An additional 899 sequences that contained MS were also obtained. Comparing the number of loci detected across different taxa is difficult because genomes vary substantially in their MS frequency. In this study, only 2.26% of all contigs obtained using 454 sequencing contained a MS. Using an identical MS isolation method, MS were detected in more than 35.2% of contigs in the soybean aphid *Aphis glycines* [22], more than 16.8% of contigs in the water strider *Gerris incognitus* [21] and more than 4.6% of contigs in the bream *Megalobrama pellegrini* [23]. Therefore, we detected fewer loci here than in other studies, indicating that MS are relatively infrequent in *H. diversicolor supertexta*. The number of MS detected is likely to be inflated by the occurrence of multiple reads covering the same sequence [21]. The increased use of high-throughput sequencing technology for marker development will allow for the exploration of factors influencing the abundance and type of MS across taxa in the future.

Additionally, the cross-species amplification of 20 MS markers was performed in another subspecies, *H. diversicolor diversicolor*. All the primer pairs effectively amplified their target sequences using the same PCR conditions because these species are closely related based on taxonomy. Cross-species amplification is effective only if primer sequences are conserved between species, and generally, the number of amplified loci tends to decrease proportionally with an increasing divergence between species [30]. We note that the two species studied in this experiment are the most important abalone fishery resources in Jeju, Korea.

### 2.3. Genetic Variability within and between the Wild and Released Populations

Two wild and hatchery-bred populations of *H. diversicolor supertexta* (each *n* = 60) collected from the coastal waters of Donggwi (Jeju Island, Korea) were screened for variation by the 20 newly uncovered polymorphic MS loci. The 20 primers yielded variable profiles, and reruns were performed for 26.7% of each of the sample sets to ensure that the allele scoring was reproducible. No differences were observed, indicating that genotyping errors did not affect allele scoring. Samples that failed to amplify after the rerun were not included, which reduced the likelihood that our results were affected by poor DNA quality.

A MICRO-CHECKER analysis revealed that 13 loci (KHds1, KHds3, KHds4, KHds5, KHds8, KHds9, KHds11, KHds13, KHds14, KHds16, KHds18, KHds19 and KHds20) could have been influenced by one or more null alleles in the wild and released samples; our data demonstrated that seven loci (KHds2, KHds3, KHds4, KHds8, KHds9, KHds18 and KHds20) were affected by null alleles in both samples, an effect that could be problematic for population genetic analyses that assume Hardy-Weinberg equilibrium (HWE). The global outlier test using both populations with LOSITAN under both infinite allele and stepwise mutation models detected two loci (KHds8 and KHds9) that had an excessively high or low *F*_ST_ relative to neutral expectations, indicating that these loci may be subject to selection. To minimize the detection of false positives, we either assumed that null alleles were present at these seven loci or that they were under directional selection because they were detected by both methods with high statistical support. Thus, these loci were eliminated from subsequent analyses.

All 20 MS loci were highly polymorphic in both populations. A total of 243 different alleles were observed and the average number of alleles per locus was18.7. The number of alleles varied from eight at the KHds6 locus to 39 at KHds14 (Table 2). Not all loci were equally variable; for example, KHds5 and KHds14 displayed greater allelic diversity and higher levels of heterozygosity. The observed heterozygosity ranged from 0.328 at the KHds6 locus to 0.938 at KHds12, whereas the expected heterozygosity varied from 0.369 at KHds6 to 0.955 at KHds14 (Table 2). The wild population had a higher number of alleles than the released population, but this difference was not significant (Wilcoxon signed-rank test, *p* > 0.05). The mean observed and expected heterozygosities in the wild samples were 0.809 and 0.814, respectively, and the corresponding parameters in the hatchery-bred released samples were 0.697 and 0.801, respectively. There were no significant differences in expected and observed heterozygosities between the wild and released populations (Wilcoxon signed-rank test, *p* > 0.05).

In this study, a high level of genetic diversity (mean heterozygosity = 0.81; mean allelic number = 15.9) in the wild population was detected. A similarly high genetic diversity has been reported in many other marine bivalves [31,32] including small abalone [12], indicating that this is a common characteristic of bivalves. Large population sizes and high nucleotide mutation rates are possible explanations for this high level of diversity [33].

Inbreeding coefficients (*F*_IS_) varied among the markers from −0.040 (KHds16) to 0.515 (KHds31) in the released samples and from −0.131 (KHds7) to 0.108 (KHds19) in the wild samples. The average *F*_IS_, including all markers, was 0.123 in the released samples and 0.013 in the wild samples (Table 2).

The allele frequency distributions were varied between the wild and released populations. However, the results of a heterogeneity test of allele frequency distributions in wild and released samples showed no significant heterogeneity across all loci after sequential Bonferroni correction for multiple tests (*p* < 0.004), indicating no distinct differences. Similar unique alleles were observed in the wild and released populations (45 *vs*. 44). Allele frequency distributions indicated that 48.6% (118/243) of alleles in the released sample were rare alleles (frequency <5%), whereas 50.6% (123/243) of alleles in the wild sample were rare alleles. Rare alleles were detected at most loci in both populations. An examination of pair-wise linkage disequilibrium revealed that all 20 MS loci were in linkage equilibrium (*p* > 0.004).

To examine the departure from mutation-drift equilibrium based on heterozygosity excess (or deficiency) in both populations, a bottleneck analysis was conducted using Bottleneck software under a two-phase model of MS mutation. None of the populations displayed a significant heterozygosity excess (*p* > 0.05) using either the sign test, standardized differences test or the Wilcoxon signed-rank test, suggesting that neither the wild nor released populations have experienced a recent bottleneck.

Significant deviations from HWE after Bonferroni correction (*p* < 0.004) were found at six loci (KHds1, KHds5, KHds10, KHds11, KHds13 and KHds14) in the released samples, and all were deviations from HWE due to heterozygote deficiency.

In released populations, homozygote excess is commonly caused by founder effects. Significant heterozygote deficiency has previously been reported in marine invertebrate species [34,35], and null alleles are the most likely cause of the heterozygote deficiency in HWE tests [36]. Indeed, our MICRO-CHECKER analysis suggested the presence of null alleles at the KHds5, KHds11, KHds13 and KHds14 loci with a significant heterozygote deficit. Thus, the homozygote excess observed in the released population was likely caused by null alleles, which is a locus-dependent effect frequently observed at MS. The widespread occurrence of null alleles has also been reported in other marine bivalves [37].

### 2.4. Genetic Variability between the Wild and Released Populations

Single-locus *F*_ST_ estimates and global multi-locus *F*_ST_ values were not different between the hatchery and wild populations. When the KHds2, KHds3, KHds4, KHds8, KHds9, KHds18 and KHds20 loci were excluded, the global multi-locus *F*_ST_ was estimated at 0.002 (*p* > 0.05).

The analysis of molecular variance (AMOVA) of the 13 MS loci yielded results similar to those from the FSTAT analysis; variation within individuals, among individuals within populations, and among populations was 93.1% (*p* = 0.000), 6.74% (*p* = 0.000), and 0.2% (*p* = 0.960), respectively (Table 3). The AMOVA indicated that the variation between the wild and released populations explained only 0.2% of the total variance, whereas the variation among individuals within populations and the variation within individuals explained 6.74% and 93.1% of the total variation, respectively.

Statistical analysis of the fixation index (*F*_ST_) and analysis of molecular variance (AMOVA) indicated no significant genetic differentiation between the wild and released populations. However, the genetic difference was confirmed by the genetic diversity parameters for each population mentioned above. In the *H. diversicolor supertexta* populations of Donggwi (Jeju Island, Korea), the seeds released for stock abundance had a different genetic composition, although no significant reductions in diversity were observed compared with the wild population (*p* > 0.05; Table 2). This difference is likely a result of hatchery selection and inbreeding. Continued hatchery reproduction might lead to considerable decreases in genetic variability [38,39]. The limited genetic differentiation between the wild and released populations suggests a relatively short release history of hatchery-bred abalone.

Monitoring the genetic diversity of wild and released populations of *H. diversicolor supertexta* in relation to stock enhancement in Jeju Island, Korea, is vital for stock abundance recovery and for planning sustainable fishery management. However, this study was limited by the number of populations screened. The genetic diversity parameters for each population may be explained using data from additional populations, which may allow for a more precise genetic characterization of the MS loci used. Therefore, our results should be interpreted with caution. Further research is required to assess the genetic resources of wild populations and the influence of stock enhancement practices on the genetic structure of this important fishery species.

## 3. Experimental Section

### 3.1. Sample Collection and 454 Sequencing

One wild and one released population of *H. diversicolor supertexta* (each *n* = 60; shell length, 4–5 cm) were sampled from the coastal waters of Donggwi (Jeju Island, Korea) in May and June of 2010. Many hatchery-raised seeds (2 cm shell length, 12–14 months of age) have been released in this water since 2007 as part of a stock enhancement program begun by the JPFI. Hatchery-raised abalone seeds were produced in part from the brood stock captured and reared for reproduction at the JPFI and purchased by the Jeju Special Self-Governing Province. No details regarding the founding and maintenance of the purchased hatchery-raised seeds are available; however, their original parents were maintained at a farm on the coast of Jeju, Korea. Samples were selected by experienced researchers based on the shell characteristics of wild animals; for released specimens, a clear green shell coloration 2 cm in length on the top of the shell (typically observed in hatchery-raised abalone) and/or metal tags on the shells (which were applied as part of the stocking practice) were also used for identification. We considered the impact of stocking on the wild population to be negligible in the case of abalone with shell lengths over 5 cm (4–5 years of age). For cross-amplification, eight individuals of *H. diversicolor diversicolor* were collected from the coast near Donggwi on Jeju Island, Korea, in March 2011. Species identification was verified by morphological distinctions [40]. Tissue samples (approximately 1 cm^3^) were placed in absolute ethanol and kept frozen at −20 °C until DNA extraction.

For MS isolation, the TNES-urea buffer method [41] was used to isolate high-molecular-weight DNA (≥2 μg) from the mantle musculature tissue of an individual abalone of *H. diversicolor supertexta*. A whole-genome shotgun library was generated from 2 μg of genomic DNA with the GS DNA Library Preparation Kit (Roche Applied Science: Indianapolis, IN, USA) according to the manufacturer’s protocol. The DNA library was titrated by sequencing using the Genome Sequencer FLX system (Roche Applied Science: Indianapolis, IN, USA). Based on the results of the titration sequencing run, an appropriate amount of the DNA library was used to set up the emulsion PCR. Subsequently, the clonally amplified DNA fragments bound to the capture beads were enriched and sequenced on 1/8 of a plate in a Genome Sequencer FLX Titanium instrument (454 Life Sciences Corp., Roche Applied Science: Indianapolis, IN, USA).

To characterize the MS loci via genotyping, total DNA from sections of the mantle musculature of each abalone sample was extracted using a MagExtractor-Genomic DNA Purification Kit (Toyobo: Osaka, Japan) for an automated DNA extraction system, the MagExtractor MFX–2100 (Toyobo: Osaka, Japan). The extracted genomic DNA was stored at −20 °C until further use.

### 3.2. Microsatellite Discovery and Primer Screening

The resulting raw sequences from *H. diversicolor supertexta* were assembled into contigs using Newbler software, verion 2.3 (454 Life Sciences: Branford, CT, USA). A PERL script was run to select sequences longer than 300 bp with a minimum of five repeats of di-, tri- or tetra-nucleotide repeat motifs. Of the reads that were identified, a subset that had a minimum of seven repeats was selected. For these reads, a PERL script was also used to design the primers, and the following criteria were used to identify loci that could be reliably amplified: (i) a GC content of 30%–90%; (ii) a product size of 90–250 bp; (iii) a primer length of 18–20 bp; and (iv) a melting temperature of 58–68 °C. Primer redundancy was tested using NCBI BLAST [42].

### 3.3. DNA Amplification and Genotyping

The newly designed PCR primer pairs were tested for consistency in PCR amplification and polymorphisms; these tests were performed on a sample set of eight abalones collected from Donggwi on Jeju Island, Korea. PCR amplifications were performed using an ABI 9700 Thermal Cycler System (Applied Biosystems) in 25 μL solution containing 12.5 μL of 2× Multiplex PCR Pre-Mix (SolGent: Daejeon, Korea; Cat. No. SMP01-P096), 100 ng of template DNA and 10 pmol of each primer. The forward primer from each pair was 5′-end-labeled with 6-FAM and HEX dyes (Applied Biosystems). PCR reactions were run for 15 min at 95 °C followed by 30 cycles of 20 s at 95 °C, 40 s at 54 °C, and 2 min at 72 °C with a 3-min final extension at 72 °C. The annealing temperature (54 °C) was 4 °C–5 °C below the Tm estimated from the nucleotide compositions of the primer pairs served as the annealing temperature. The PCR amplification was considered to be successful based on the presence of a visible band after running 5 μL of the PCR product on a 5% denaturing agarose gel. The 1 kb Plus DNA Ladder molecular weight marker (SolGent: Daejeon, Korea; Cat. No. SDL54-B500) was used as a standard to assess the product size. If no amplification was detected, that primer set was excluded from further analysis by multiplex PCR. Some loci could not be amplified from any of the samples, and some yielded only faint bands even after adjustment of the PCR conditions. We excluded these loci from further testing. For the remaining loci, genetic variation was examined in all samples collected. MS polymorphisms were identified using an ABI PRISM 3100 Automated DNA Sequencer (Applied Biosystems), and alleles were designated by PCR product size relative to a molecular size marker (GENESCAN 400 HD [ROX], Applied Biosystems). Fluorescent DNA fragments were analyzed using GENESCAN, version 3.7 (Applied Biosystems Inc., Foster City, CA, USA) and GENOTYPER, version 3.7 (Applied Biosystems Inc., Foster City, CA, USA) software packages (PE Applied Biosystems). The populations were multiplexed for genotyping by pooling samples tagged with different dyes within a well. We assessed the reliability of the primers by repeating the amplification and genotyping 16 samples from each population (26.7%).

Finally, all of the newly developed polymorphic MS loci in *H. diversicolor supertexta* were assessed for cross-amplification in another congener species, *H. diversicolor diversicolor*, using eight individuals.

### 3.4. Population Comparisons

Populations were screened for variation at the newly developed MS loci. MICRO-CHECKER 2.2.3 [43] was used to detect genotyping errors due to null alleles, stuttering, or allele dropout using 1000 randomizations. We also determined whether one or more MS were under selection by examining the data using LOSITAN software [44], which is a selection-detection workbench based on a well-evaluated *F*_ST_-outlier detection method. To access genetic diversity, the number of alleles per locus (*N*_A_), size of alleles in base pairs (*S*), frequency of the most common allele (*F*), and number of unique alleles (*U*) were determined for each local population at each locus using the GENEPOP program, version 4.0 [45]. GENEPOP was also used to identify deviation from HWE (exact tests, 1000 iterations) as well as the observed and expected heterozygosities, which indicates an excess or deficiency of heterozygotes. FSTAT version 2.9.3.2 [46] was used to calculate the inbreeding coefficient (*F*_IS_) [47] per locus. For AMOVA [48], components of variance within and between populations based on the infinite allele model (IAM) were estimated using the ARLEQUIN program, version 3.0 [49]. The significance of AMOVA components was tested using 1000 permutations. ARLEQUIN was also used to calculate single-locus and global multilocus values (*F*_ST_; 1000 permutations) [47] and to assess linkage disequilibrium for all pairs of loci, the empirical distribution of which is obtained by a permutation procedure [50]. Significance levels were adjusted for multiple tests using a sequential Bonferroni correction [51]. Because hatchery populations are often subjected to founder effects and bottlenecks that result in decreased genetic diversity, Bottleneck software, version 1.2.02 [52] was employed to test the bottleneck hypothesis under a two-phased model of mutation (TPM). This method can be used for testing the departure from mutation-drift equilibrium based on heterozygosity excess or deficiency.

## 4. Conclusions

In conclusion, despite the release of hatchery-bred seeds into natural coastal areas to compensate for the reduced fishery resources in Korea, to date, no detailed genetic information is available on the genetic diversity of wild and released stocks of *H. diversicolor supertexta*. In this study, we report the development of 20 polymorphic MS markers for *H. diversicolor supertexta* using 454 pyrosequencing. This study is among the first few studies to validate the next-generation sequencing method of MS acquisition for a commercially important fishery species. No significant genetic difference was detected between the wild and released populations based on an analysis of 13 MS loci, with none of null alleles being detected in either population. Continued monitoring of the genetic variance between wild and released abalone stocks using DNA markers is essential for the successful implementation of an artificial enhancement project and the conservation of natural Korean *H. diversicolor supertexta* resources. Moreover, primers for all polymorphic loci can be used in the related species *H. diversicolor diversicolor*.

## Figures and Tables

**Table 1:** Characteristics of the 20 microsatellite loci developed for *Haliotis diversicolor supertexta* and their cross*-*amplification in *Haliotis diversicolor diversicolor*.

Locus	Repeat motif	Primer sequence (5′-3′)	*T*a (°C)	Cross-amplification in *H. diversicolor diversicolor*	GenBank Accession No.
KHds1	(GCT)_8_	F: **HEX-**CTCAAAGTCTTGAAAGGTTCT R: AGGTGTCAAGTAGTCCTGAAA	54	+	JQ678720
KHds2	(CA)_7_(AT)_5_	F: **FAM-**ACGCCCTGTTACTTCTGATCC R: GACTGAAGTAAGTACTCGTGT	54	+	JQ678721
KHds3	(CTCA)_8_	F: **HEX-**GTCTGTGATGAAATGTAGTCT R: GAGATTACAACAGGTGATTGC	54	+	JQ678722
KHds4	(GTAA)_10_	F: **HEX-**CACGACACACAAATGATCATT R: GTTTCTGACAAAGTACTCGAG	54	+	JQ678723
KHds5	(CCGCA)_19_	F: **HEX-**CTCCGCTCTCCGGGCGGACGC R: CCATATCAGACTTCACGCTGTAATC	54	+	JQ678724
KHds6	(GATT)_9_	F: **HEX-**GGACTGATCTGTGGTAGAGCT R: GGAGACTACTATTCTCATGGA	54	+	JQ678725
KHds7	(CA)_8_	F: **FAM-**GGTATAATTCTCACATGCACG R: ATCTCATCGACTGGAGGGACA	54	+	JQ678726
KHds8	(GTGA)_8_	F: **HEX-**GCAGGGACACCTGATTTGAAC R: GCCTTTGCATGGTATGATCTA	54	+	JQ678727
KHds9	(GATA)_9_	F: **FAM**-ACTAGCAGTAACTGCGACACC R: GATCTCGGCACATGACTATAT	54	+	JQ678728
KHds10	(TCA)_9_	F: HEX-TCGTTAACATTCCCCGGAAAT R: ATCGAGGAAAATACCAGCGCC	54	+	JQ678729
KHds11	(TGAG)_8_	F: **FAM-**CCTGCAATCAGCATATCAAAA R: CTTACAGTGATACTGCTGGAT	54	+	JQ678730
KHds12	(CTA)_11_	F: **FAM-**TGGTATTCGTTACTACTACTAC R: GACATGGACATAGATAGACAA	54	+	JQ678731
KHds13	(CCTCA)_10_	F: **HEX-**AGCTAAACATTCTCAAGCACG R: GGCTATACTTCATAACCGATG	54	+	JQ678732
KHds14	(TTTA)_14_	F: **HEX-**CTAATTTACCCAGACTGTAAC R: AGTTACACCTCACCTTACCAA	54	+	JQ678733
KHds15	(CATT)_9_	F: **FAM-**TTGGCCTAATTTGGAAGCTAG R: CAATATGAGTCAGGTTAGATC	54	+	JQ678734
KHds16	(TA)_8_	F: **FAM-**GCGCCCAAAAAACCTGTCCCA R: TCAGCTGCCTGTTCTATTCAC	54	+	JQ678735
KHds17	(CA)_8_	F: **FAM-**AACCTAACATCCACGCACACG R: CAGTCTCCGAAACCCTATAAT	54	+	JQ678736
KHds18	(GTGGGT)_8_	F: **HEX-**CGGTGGTTGACTGTGTGTTGG R: CACAAGGACATTTAGTGGTTGA	54	+	JQ678737
KHds19	(AC)_10_	F: **FAM-**CGAAATCACACTTCATGCATT R: TGAAGACCACATGTGTGTGTG	54	+	JQ678738
KHds20	(CTGT)_8_	F: **HEX-**GAGCTGATCAAATGTGTAGTA R: AGTCTGGACTATACAATCCAG	54	+	JQ678739

*T*_a_ is the optimal annealing temperature; + means that it did cross-amplify.

**Table 2:** Summary of the statistics for 13 microsatellite loci in two populations of *Haliotis diversicolor supertexta*.

Microsatellite loci	*F_S_*_T_	Population (No.)															

		Wild (60)								Released (60)							

		*N*_A_	*S*	*F*	*U*	*H*e	*H*o	*F*_IS_	*p*	*N*_A_	*S*	*F*	*U*	*H*e	*H*o	*F*_IS_	*p*
	
KHds1	−0.0026	9	100–136	0.627	2	0.590	0.593	−0.005	0.494	11	100–136	0.678	0	0.530	0.475	0.105	0.002 ^*^
KHds5	0.0055	26	114–249	0.186	6	0.919	0.898	0.023	0.369	25	117–243	0.110	7	0.936	0.847	0.095	0.000 ^*^
KHds6	0.0100	7	106–142	0.678	1	0.504	0.525	−0.044	0.673	6	106–124	0.784	2	0.369	0.328	0.113	0.011
KHds7	−0.0010	21	110–169	0.517	6	0.717	0.810	−0.131	0.923	18	110–169	0.483	5	0.736	0.746	−0.013	0.901
KHds10	−0.0051	12	96–130	0.220	1	0.851	0.915	−0.076	0.772	12	100–130	0.203	1	0.859	0.695	0.193	0.003 ^*^
KHds11	0.0032	19	118–202	0.164	3	0.916	0.845	0.078	0.004	17	118–191	0.161	5	0.907	0.831	0.085	0.000 ^*^
KHds12	−0.0023	17	186–249	0.169	2	0.908	0.915	−0.008	0.006	16	186–255	0.188	3	0.902	0.938	−0.040	0.158
KHds13	0.0012	11	102–152	0.216	1	0.863	0.914	−0.059	0.191	10	92–142	0.259	2	0.840	0.569	0.325	0.000 ^*^
KHds14	0.0078	32	100–222	0.229	7	0.911	0.864	0.052	0.140	31	100–218	0.129	8	0.955	0.466	0.515	0.000 ^*^
KHds15	0.0033	13	138–244	0.300	3	0.817	0.883	−0.083	0.607	11	138–196	0.314	5	0.817	0.804	0.016	0.148
KHds16	0.0046	13	90–144	0.288	7	0.831	0.746	0.104	0.454	17	84–144	0.297	3	0.842	0.729	0.136	0.125
KHds17	0.0017	15	102–140	0.207	5	0.872	0.828	0.051	0.321	14	104–134	0.225	2	0.863	0.833	0.035	0.172
KHds19	−0.0040	11	120–146	0.183	1	0.878	0.783	0.108	0.081	9	120–138	0.220	1	0.857	0.797	0.071	0.176
Mean	0.0016	15.8		0.306	3.5	0.814	0.809	0.013		15.2		0.312	3.4	0.801	0.697	0.123	

The single-locus *F*_ST_, number of samples (No.); number of alleles per locus (*N*_A_); size of an allele in bp (*S*); frequency (*F*) of the most common allele; number of unique alleles (*U*); expected heterozygosity (*H*e); observed heterozygosity (*H*o); inbreeding coefficient (*F*_IS_); and probability of significant deviation (^*^) from Hardy-Weinberg equilibrium after Bonferroni correction (*p*, initial α = 0.05/13 = 0.004) are given for each population and locus; Calculations assume that individuals with one microsatellite band are homozygous for the allele.

**Table 3:** Results of the analysis of molecular variance (AMOVA) of 13 microsatellite loci in the wild and hatchery populations of *Haliotis diversicolor supertexta*.

Source of Variation	Sum of Squares	Variance Components	Percentage Variation (%)	*p*-Value
Among populations	6.60	0.009	0.2	0.960
Among individuals within a population	640.5	0.35	6.74	<0.001
Within individuals	568.0007	4.90	93.1	<0.001
Total	1215.04	5.26		
